# Potential return on investment of a family-centered early childhood intervention: a cost-effectiveness analysis

**DOI:** 10.1186/s12889-017-4805-7

**Published:** 2017-10-10

**Authors:** Negin Hajizadeh, Elizabeth R. Stevens, Melanie Applegate, Keng-Yen Huang, Dimitra Kamboukos, R. Scott Braithwaite, Laurie M. Brotman

**Affiliations:** 1 Department of Medicine, Zucker School of Medicine at Hofstra/Northwell, 300 Community Drive, Manhasset, NY 11030 USA; 2 Department of Population Health, New York University School of Medicine, 227 E. 30th St, New York, NY 10016 USA

**Keywords:** Markov model, ParentCorps, Childhood, Obesity, Behavior problems

## Abstract

**Background:**

ParentCorps is a family-centered enhancement to pre-kindergarten programming in elementary schools and early education centers. When implemented in high-poverty, urban elementary schools serving primarily Black and Latino children, it has been found to yield benefits in childhood across domains of academic achievement, behavior problems, and obesity. However, its long-term cost-effectiveness is unknown.

**Methods:**

We determined the cost-effectiveness of ParentCorps in high-poverty, urban schools using a Markov Model projecting the long-term impact of ParentCorps compared to standard pre-kindergarten programming. We measured costs and quality adjusted life years (QALYs) resulting from the development of three disease states (i.e., drug abuse, obesity, and diabetes); from the health sequelae of these disease states; from graduation from high school; from interaction with the judiciary system; and opportunity costs of unemployment with a lifetime time horizon. The model was built, and analyses were performed in 2015–2016.

**Results:**

ParentCorps was estimated to save $4387 per individual and increase each individual’s quality adjusted life expectancy by 0.27 QALYs. These benefits were primarily due to the impact of ParentCorps on childhood obesity and the subsequent predicted prevention of diabetes, and ParentCorps’ impact on childhood behavior problems and the subsequent predicted prevention of interaction with the judiciary system and unemployment. Results were robust on sensitivity analyses, with ParentCorps remaining cost saving and health generating under nearly all assumptions, except when schools had very small pre-kindergarten programs.

**Conclusions:**

Effective family-centered interventions early in life such as ParentCorps that impact academic, behavioral and health outcomes among children attending high-poverty, urban schools have the potential to result in longer-term health benefits and substantial cost savings.

## Background

ParentCorps is a family-centered enhancement to pre-kindergarten (pre-k) programming that aims to promote family engagement and safe, nurturing and predictable environments at home and at school. When implemented in high-poverty, urban schools serving primarily Black and Latino pre-k students, it has been found to yield robust and sustained benefits through age 8 across domains of academic achievement, behavior problems, and obesity [1–5]. There is a substantial body of developmental and experimental evidence that early childhood learning, behavior and health problems cascade to predict costly and impairing life-long disorders and conditions. We sought to estimate the long-term cost-effectiveness of ParentCorps for children attending pre-k programs in high-poverty, urban schools.

Two randomized controlled trials (RCTs) in 18 high-poverty, urban schools with more than 1200 Black and Latino children provide the evidentiary foundations for ParentCorps’ impact on child health and development [1–5]. The second trial enrolled nearly 90% of the pre-k population (*n* = 1050) and intent-to-treat analysis documents impact through second grade on mental health (behavior and emotional problems) and academic achievement (teacher-rated performance and reading achievement test scores) across baseline levels of self-regulation in pre-k (e.g., impulsivity, inattention, hyperactivity). In addition, among the subgroup of pre-k students with low self-regulation (~25% of the pre-k population in high-poverty, urban schools), ParentCorps in pre-k led to substantially lower rates of obesity (defined as BMI ≥ 95th percentile) and sedentary behavior through second grade [2–5].

Longitudinal and experimental studies that follow children from early childhood into adulthood provide strong support for a cascading developmental model to explain drug abuse, antisocial behavior and interaction with the judiciary system. For example, a large prospective study of public school children [6] identified a developmental pathway starting in early childhood and resulting in substance abuse in 12th grade. Children who experienced seven risk factors over time (i.e., poverty, low self-regulation in early childhood, early parenting problems, early behavior problems, early peer problems, adolescent parenting problems, and adolescent peer problems) had a 91% chance of using illicit substances by 12th grade, compared with a population base rate of 51%. An experimental study in high-poverty, urban schools found that intervention early in elementary school prevented poor health outcomes. By ages 19 to 21, boys receiving the intervention, particularly those who entered school with behavior problems, reported significantly reduced rates of tobacco use, substance use problems and antisocial personality disorders [7].

Although there are no long-term follow-up studies of early childhood obesity prevention, children who are overweight or have obesity in early childhood are five times more likely to be overweight or have obesity as adults [8]. Therefore, an intervention such as ParentCorps that effectively reduces rates of childhood obesity and sedentary behavior would be expected to have long-term impact on adult obesity and related health behaviors [4]. In fact, three recent, independent mathematical simulation models (cost-effectiveness analyses) found that early obesity reduction in childhood and adolescence would be cost effective, due to reductions in the number of adults with obesity, lifetime medical costs and increases in quality-adjusted life years (QALYs) by the age of 40 [9–11].

Based on ParentCorps’ documented effects on academic achievement, behavior problems, and obesity, and a substantial developmental literature, we designed a mathematical model to project the impact of ParentCorps as an enhancement to pre-k in high-poverty, urban schools on life-long costs and health (life expectancy and QALYs), as compared to standard pre-k programming.

## Methods

We designed a Markov model using TreeAgePro software [12] to represent the lifespan of an individual transitioning from the end of the pre-k year (~ age 5) through childhood, adolescence and adulthood, following either exposure to ParentCorps in pre-k, or standard pre-k programming. A hypothetical child enters the model at age 5 years and, after exposure or no exposure to ParentCorps, transitions through different possible scenarios year by year after graduation from high school and into adulthood until death. What happens to the individual from childhood through adulthood is aggregated into a calculation of life expectancy and quality of life. QALYs are the most commonly used form of health-adjusted life years, [13] which encapsulate the idea that a year spent in good health is fundamentally worth more than a year spent in poor health. The model was used to simulate 100,000 hypothetical individuals and has a lifetime time horizon. The model was built and analyses were performed in 2015–2016.

### Model structure

**Fig. 1:**
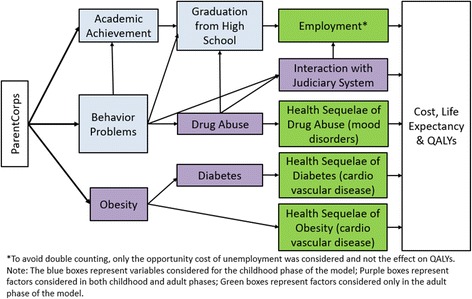
Influence diagram. Influence diagram showing the constructs embedded in the mathematical model

The structure of the model was guided by an influence diagram that reflects interrelationships of important constructs found to be changed by ParentCorps in childhood, and may be impacted by ParentCorps in adolescence and adulthood (Fig. 1). Specifically, ParentCorps promotes academic achievement and prevents behavior problems across all levels of pre-k self regulation and prevents obesity among children with low self-regulation [2–4]. The anticipated cascading effects of these documented childhood benefits are represented in the model. The model is divided into a childhood phase (including adolescence), which ends at age 18 (typical age of graduation from high school), and an adulthood phase, which follows the individual until death. The childhood phase records whether the youth develops behavior problems, abuses drugs, develops obesity, develops diabetes, achieves academically, and graduates from high school. The youth then transitions into adulthood and has a subsequent trajectory influenced by the childhood pathway.

The model is a state-transition simulation that employs “Markov states,” which represent different ways of “being” in a given time cycle. The Markov states include the combination of different health states (i.e., obesity, diabetes, and drug abuse) and their health sequelae (i.e., cardiovascular disease and psychiatric disorders); whether the hypothetical person is employed; and whether the person has an interaction with the judiciary system in a given year. To keep the model tractable and focused on the predicted longer-term effects of early intervention into adulthood, the childhood phase has only one Markov state and a time cycle of 13 years (i.e., pre-k through end of high school). The adult phase has 32 Markov states and a time cycle of 1 year. These Markov states in adulthood depend on five attributes: 1) obesity and/or health sequelae of obesity; 2) diabetes and/or health sequelae of diabetes; 3) drug abuse and/or health sequelae of drug abuse; 4) interaction with the judiciary system; and 5) employment.

The model was designed to emphasize three particularly important pathways in the influence diagram: 1) lack of basic academic proficiency (achievement) may lead to decreased employment; 2) behavior problems may lead to drug abuse and its health sequelae, and interaction with the judiciary system; and 3) obesity may lead to diabetes and its health sequelae. In the model, academic proficiency and behavior problems influence the likelihood of graduating from high school, which ultimately impacts the likelihood of employment. Behavior problems also influence the likelihood of drug abuse. Together with drug abuse, behavior problems also influence the likelihood of interaction with the judiciary system. The model represents whether a child with low self-regulation in pre-k achieves academic proficiency, develops behavior problems and/or obesity, and the impact of ParentCorps in promoting academic achievement and preventing behavior problems and/or obesity, especially among children with low self-regulation. The increased likelihood of youth with obesity developing type-2 diabetes, and its long-term health sequelae (e.g., cardiovascular disease) are also reflected in the model. Finally, employment, interaction with the judiciary system, and health sequelae of drug abuse and/or diabetes influence the outcomes that are tracked by the model (i.e., costs, life expectancy, and quality-adjusted life expectancy).

### Data inputs

Data inputs (Table 1) were obtained from the peer-reviewed scientific literature including reports by Brotman and colleagues from RCTs of ParentCorps’ impact in childhood [1–5]. Where available, odds and odds ratios were the preferred metric used to determine likelihoods of events. When these metrics were not directly available, they were back-calculated from incidence rates and/or prevalence estimates, assuming uniform incidence rates over time. Plausible ranges for each estimate were determined by the 95 % confidence intervals, or, if unavailable, by consulting with content area experts. Where necessary, we used decision rules to pool relevant data using the random effects method of DerSimonian and Laird, and tested for homogeneity defined as a Q-statistic of >0.10, I-statistic of <25% and a *p*-value of <0.05 with no significant outliers on the Forest plot [14]. If data were not homogeneous, the median value was used and the plausible range included the lowest and highest reported confidence intervals. Mortality data were obtained from the Centers for Disease Control 2008 National Vital Statistic Report and from disease specific mortality rates [15–17].

**Table 1:** Model inputs

Variable		Inputs (Odds)	Notable assumptions and explanations	Lower range	Upper range	Source population	References
Inputs influencing health							
Child Specific							
Odds ever of good academic achievement (basic academic proficiency)		1.35	Fixed prevalence throughout childhood based on a point prevalence (4th–12th grade) of 57.5% basic academic achievement	0.7	2.0	National 4th grade black public school students	[33]
Odds ever of obesity as a child (BMI at or above 95%)		0.146	Assumed a cumulative incidence of 23.8% by age 10 (5th grade) based on a point prevalence of 23.8% (K-5th grade)	0.09	0.3	NYC public elementary school students	[34]
Odds ever of low self-regulation		0.33	Fixed prevalence throughout childhood based on a period prevalence of 25%	0.15	0.4	High risk children followed infancy through grade 3	[5, 28]
Odds ever that a child will behavior problems		0.275	Fixed prevalence throughout childhood based on a period prevalence of 21.6%	0.14	0.4	Kindergarten or first grade students given any early poverty	[29]
Odds of graduating from high school		2.125	One-time event, estimated from incidence of 68%	1.59	2.66	Low-income New York state high school students	[35]
Odds of any drug abuse disorder		0.13	Fixed prevalence throughout childhood based on a point prevalence of 11.4%	0.11	0.15	Nationally representative face-to-face survey of adolescents aged 13 to 18 years in the continental United States.	[50]
Odds ever of type 2 diabetes as a child		0.001	Fixed prevalence throughout childhood based on a point prevalence of 1.05 per 1000	0	0.002	Black children aged 0–19 with type 2 diabetes	[37]
Odds ever of interacting with the judiciary system as a child (likelihood of one violent crime arrest)		0.099	Fixed prevalence throughout childhood based on a point prevalence of 9%	0.074	0.124	Low-income urban Baltimore adolescents	[24]
Odds ratio good academic achievement given child received ParentCorps		1.520		1.000	2.430	ParentCorps	[23]
Odds ratio good academic achievement given child has behavior problems		0.229		0.084	0.621	ParentCorps	[23]
Odds Ratio child becomes obese given they have low self-regulation in pre-k		3.846		1.36	4.50	ParentCorps	[4]
Odds Ratio child becomes obese given they have low self-regulation in pre-k and received ParentCorps		0.260		0.080	0.865	ParentCorps	[4]
Odds Ratio child becomes obese given that they do not have low self-regulation and received ParentCorps		1.000		0.82	1.0	ParentCorps	[4]
Odds Ratio child develops behavior problems given they received ParentCorps		0.590		0.41	0.85	ParentCorps	[1, 3, 5]
Odds Ratio child develops behavior problems given low self-regulation		3.800		2.0	5.5	Urban children from Arizona aged 55–97 months	[1, 3, 5, 20]
Odds Ratio child graduates from high school given they have good academic achievement		1.335		1.0	4.0	Children from Tennessee’s Project STAR evaluating graduation among children in K-3 grade (55.8% free lunch)hn	[22]
Odds Ratio child graduates from high school given they abuse drugs		0.699		0.584	0.826	US national sample adults over 18 surveying back on their childhood	[19]
Odds Ratio child graduates from high school given they have behavior problems		0.180		0.1	1.0	Adolescents ages 15–20 with serious emotional disturbance, 38.2% low income, 39.5% urban	[25]
Odds Ratio child abuses drugs given they have behavior problems		3.800		1.0	5.0	New Zealand urban children	[51]
Odds Ratio child with obesity develops diabetes		5.100		1.51	17.0	Children ages 4–19 in rural Canada	[26]
Odds Ratio child interacts with judiciary system given they abuse drugs		5.700		2.30	15.05	Study among urban New Zealand adolescents with alcohol misuse and juvenile offenses	[51]
Odds Ratio child interacts with judiciary system given they have behavior problems		2.925		1.300	6.375	Study among New Zealand males with childhood onset versus adolescent onset antisocial behavior	[31]
Adult Specific							
Odds of developing obesity per yr. (BMI > 30)	Age 18–39	0.003	Constant incidence rate, based on a point prevalence of 30% for ages 20–39; 39.5% for ages 40–59; and 35.4% for age > 60; assumed to be cumulative incidence for each age range.	0	0.064	The National Health and Nutrition Examination Survey 2007–2008, a representative sample of the US population with measured heights and weights on 3281 children and adolescents (2 through 19 years of age) and 719 infants and toddlers (birth to 2 years of age).	[30]
	Age 40–59	0.005		0.001	0.01		
	Age > 60	−0.002		0	0.005		
Odds of abusing drugs as an adult per yr. (any alcohol use/dependence)		0.007	Constant incidence rate estimated based on lifetime cumulative incidence of 26.6%	0.002	0.013	Survey of psychiatric disorders among persons aged 15 to 54 years in the US noninstitutionalized civilian population	[52]
Odds of developing diabetes as an adult per yr		0.0069	Constant incidence rate assumed based on cumulative incidence of 6.9 per 1000 (age 18–79)	0.006	0.008	CDC data on incidence of diagnosed diabetes among people aged 18–79	[53]
Odds of employment		12.7	Fixed prevalence based on a point prevalence of 92.7%; assumed fixed effect throughout adulthood (i.e., if employed stay employed and if unemployed stay unemployed from yr. to yr)	9.52	15.87	National unemployment rate from August 2013	[54]
Odds Ratio adult with obesity develops diabetes		7.370		6.39	8.50	Random digit phone survey of US adults aged 18 yrs. or older participating in Behavioral Risk Factor Surveillance System in 2001	[32]
Odds of newly interacting with the judiciary system as an adult per yr. (likelihood to ever go to prison)		0.002	Constant incidence rate assumed, based on lifetime cumulative incidence of 4.5%	0	0.005	Bureau of Justice data on lifetime likelihood of going to prison	[55]
Odds of new psychiatric disorders as an adult per yr. (diagnosis of any mood disorder)		0.007	Constant incidence rate within age groups estimated based on lifetime prevalence of mood disorder starting at age 18.	0.005	0.009	Survey of US residents aged 18 yrs. and older in National Comorbidity Survey Replication 2001–2003	[56]
Odds of cardiovascular disease as an adult, per yr	Age 18–44	0.004	Constant incidence rate within each decade based on annual incidence rate	0.002	0.006	NHLBI morbidity & mortality chart book on cardiovascular, lung and blood diseases	[16]
	Age 45–54	0.007		0.006	0.008		
	Age 55–64	0.015		0.010	0.022		
	Age 65–74	0.028		0.020	0.035		
	Age 75–84	0.052		0.045	0.060		
	Age 85–94	0.075		0.07	0.08		
Odds Ratio of cardiovascular disease given that an adult has diabetes		2.300	Cardiovascular disease as the major health sequelae impacting life expectancy and quality of life calculated based on annual incidence rate of CVD in diabetes	1.5	3.5	Framingham study on cardiovascular disease and diabetes	[23]
Odds Ratio of cardiovascular disease given that an adult has obesity		2.300	Assume to be the same as risk of CVD in patients with diabetes	1.5	3.5	Framingham study on cardiovascular disease and diabetes	[23]
Odds Ratio of any psychiatric disorder given drug abuse		4.5	Any psychiatric disorder as the most significant health sequelae of drug abuse impacting life expectancy and quality of life; calculated based on lifetime co-occurrence of any mood disorder (depression, dysthymia mania) given alcohol abuse	3.36	7.38	NIMH interview of comorbid alcohol, other drug and mental health disorders; national survey of drug use and health	[57–59]
Odds Ratio adult interacts with judiciary system given they abuse drugs		4.14		1.2	5.5	Prospective study of US criminal offenders substance use drug treatment and crime	[60]
Odds Ratio adult interacts with judiciary system given interacting with the judiciary system in the last yr		227	Back calculated using the odds of interaction with judiciary within 3 years of release from prison	50	250	Prospective study of US criminal offenders’recidivism	[61]
Odds Ratio of employment given graduated high school		1.88		1.5	3.0	Bureau of labors statistics report of college enrollment and work activity of 2015 US high school graduates	[62]
Odds Ratio of employment given interacting with the judiciary system in the last yr		0.029		0.005	0.1	Study of employment among adults released from NYC jails	[63]
Odds Ratio of using drugs given a history of abusing drugs in the last yr		47.62		35	571	Study of recovery from alcohol dependence among US adults	[64]
Odds Ratio of obesity given obese in the last yr		331	Back-calculated using Odds of 0.007 of non-persistence of obesity in adults with obesity. Assumed constant incidence rate, based on cumulative incidence risk of 14% over 21 year follow up (from age 17 to 38) and fixed prevalence after age 38.			National longitudinal survey of US youths	[10, 65, 66]
Inputs influencing costs							
		Inputs ($)	Cost Unit	Lower range ($)	Upper range ($)		References
Annual drug abuse treatment cost		$1000	per person per year	500	1500		[67]
Annual drug abuse complication cost		$21,483	per person per year	10,742	32,225		[42]
Annual diabetes treatment cost		$9975	per person per year	4988	14,963		[41]
Annual diabetes complication cost		$1575	per person per year	788	2363		[40]
Annual obesity treatment cost		$0	per person per year	0	0		[68]
Annual obesity complication cost		$732	per person per year	366	1098		[43]
Annual judiciary system cost of incarceration		$28,893	per person per year	14,447	43,340		[44]
Annual unemployment opportunity cost		$33,160	per person per year	16,580	49,740		[45]
ParentCorps ongoing annual costs per child		$500	per child	N/A	N/A		
ParentCorps capacity building costs per school		$100,000	per school	N/A	N/A		
Inputs influencing utilities							
		Inputs (Utility)					References
Utility of having diabetes		0.690					[69]
Utility of having complications of diabetes		0.350					[69]
Utility of drug abuse		0.670					[70]
Utility of having complications of drug abuse		0.600					[70]
Utility of being obese		0.710					[71]
Utility of having complications of obesity		0.500					[71]
Utility of being in prison		0.725					[72]

Note: In the model odds are adjusted using odds ratios and then converted to probabilities using the formula probability = odds/(1 + odds)

### Outcomes

Outcomes modeled were: 1) costs resulting from the development of the three disease states (i.e., obesity, diabetes, or drug abuse), health sequelae of these disease states (i.e., cardiovascular disease and psychiatric disorders), interaction with the judiciary system, and opportunity cost of unemployment; and 2) QALYs, which are affected by the development of the three disease states, their health sequelae, and interaction with the judiciary system.

### Assumptions

As with all decision models, several assumptions were necessary. When there was uncertainty, we strove to be conservative, overestimating intervention costs and underestimating benefits.

#### General data assumptions

Most childhood inputs were based on data representing the demographics of youth living in urban areas in the United States [1–5, 18–37]. The following assumptions were made and tend to underestimate benefits and cost savings: The effect of behavior problems leading to drug abuse would manifest itself in the childhood phase only, and the health sequelae of diseases occurred only after youth became adults. As they were beyond the scope of these analyses, we did not consider the effect of graduation from high school on the likelihood of interaction with the judiciary system as an adult, the effect of poor health on employment, or the potential cost savings from ParentCorps due to decreased need for academic remediation, special education services, or mental health services during childhood and adolescence. Additionally, we assumed that the health sequelae with the greatest impact on life expectancy and quality of life was the development of cardiovascular disease for both diabetes and obesity, and further assumed that the likelihood of developing cardiovascular disease was the same for those with obesity and those with diabetes. This assumption was necessary due to the lack of data separating the likelihood of persons with obesity developing cardiovascular disease independent of diabetes. We did however consider the costs of other health sequelae in the total cost of diabetes and obesity health sequelae as described below.

#### Utility assumptions

“Utility” is a preference-weighted quality of life metric that is typically represented on a scale of 0 (death) to 1 (perfect health), and is used to calculate QALYs. To avoid double-counting when unemployment costs are represented, we assumed that there was no impact on utility of unemployment [38]. Utilities were assigned to the development of each of the three disease states, health sequelae of each disease, and for interaction with the judiciary system. For joint utilities having more than one disease or health sequelae, we used the minimum utility among the conditions [39].

#### Cost assumptions

We used the societal cost perspective in the model and focused on major cost drivers associated with ParentCorps implementation in a high-poverty, urban elementary school and the lowered costs associated with academic, behavior and health benefits from ParentCorps ultimately resulting in a lower likelihood of: 1) diabetes; 2) drug abuse; 3) interaction with the judiciary system; and 4) unemployment. Costs of health conditions were based on estimates from published reports [40–44]. We considered the costs of treatment for the disease state (obesity, diabetes, and drug abuse) separately from the cost of treatment for the health sequelae of the disease states (for diabetes this included costs of macrovascular disease, nephropathy, neuropathy, and retinopathy; for obesity this included the cost of treatment for hypertension, lipid disorders, coronary heart disease, and stroke; and for drug abuse this included the costs of treatment for drug abuse related psychiatric disorders).

Cost of incarceration was based on average annual judiciary system cost of incarceration by the US government [44]. The opportunity cost of unemployment was based on the hourly wage for non-farm workers [45].

ParentCorps costs were calculated by an independent consulting firm based on cost calculations from historical documentation of implementation in 18 high-poverty, urban schools participating in the two trials (2003–2011) and prospective documentation of implementation costs in 20 schools (2013–2014) (Wellspring Consulting LLC: Strategic Growth Plan for ParentCoprs in NYC, unpublished). Costs are per school and assume an average of 4 classrooms of 18 pre-k students (*n* = 72 students). Children and families participate in programming during the pre-k year only. Costs include capacity building at the school-level (e.g., group-based training, individual coaching for teachers and school-based mental health professionals) for teachers and mental health professionals to implement ParentCorps with fidelity; and annual recurring costs associated with implementing ParentCorps programs (e.g., materials and tools for parents and children, meals for families and staff pay when programming is provided after school hours) with resources to serve all pre-k children (72 annually per school) and the majority (~80%) of their parents. The cost of capacity building (and implementation) over the first 2 years is $200,000, and is conservatively estimated to last for 5 years before requiring substantial additional investment. The annual cost of implementation is $40,000 (see appendix for table that breaks down these costs). Therefore, over a 5-year period, it costs $320,000 to serve 360 (72 × 5) pre-k students. This results in $888 in program expenditures per student. In sensitivity analyses, we varied assumptions regarding these costs, including the number of pre-k students per school (best case: 100 students; worst case: 1 student), the percent of pre-k students with low levels of self-regulation (best case: 30% of students; worst case: 15% of students) and how long the investment in capacity building (training and coaching) would last (best case: 10 years; worst case: 1 year). We employed a conservative discount rate of 5%, reflecting the idea that a cost or benefit in the future is valued less than the same cost or benefit today [39]. All costs are reported in 2015 $US.

### Base case scenario

In the base case scenario, we assumed 72 pre-k students per school and the initial capacity building investment lasting 5 years. We assumed that 25% of students entered pre-k with low levels of self-regulation as is typically found in high-poverty schools [1–5, 28].

### Sensitivity and threshold analyses

Sensitivity analysis tests the degree to which the model’s outcomes are affected by changes in data inputs across plausible ranges. Specifically, each input is changed individually across its plausible range to test the effect of this change on the model’s outcomes. In addition, we performed threshold analyses for variables identified in one-way sensitivity analyses as having large effect on downstream costs and/or utility, in order to identify the thresholds at which estimates for these variables would change the model’s key inferences for decision making, even if their plausibility was low.

## Results

In the base case scenario, ParentCorps was both cost saving and improved health over the life course. ParentCorps saved $4387 per person in healthcare, criminal justice, and productivity expenditures, after factoring in program costs (spending $888 to save $5275 per person over the long-term). ParentCorps increased each individual’s quality-adjusted life expectancy by 0.27 QALYs.

### Validation

We compared the model’s predictions for life expectancy to the life expectancy reported for adults with obesity and those of normal weight from the National Death Index [46]. Our model estimates a life expectancy of 60.1 Life Years for an 18-year-old with obesity, which is within the range of life expectancy from the National Death Index which reports life expectancies for 18-year-old individuals with obesity to be 57.5 Life Years.

### Sensitivity analyses

**Fig. 2:**
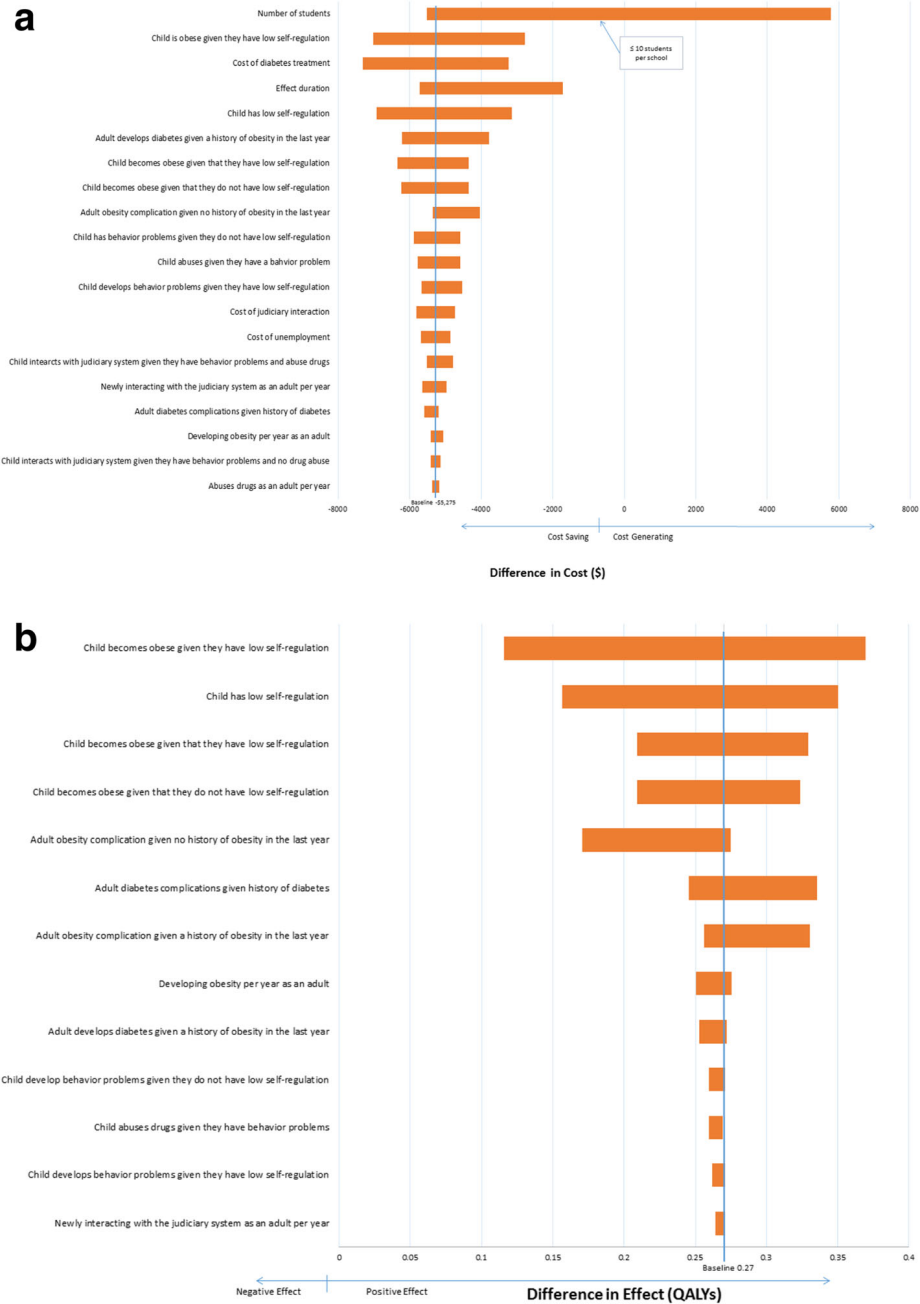
Sensitivity analyses effect on costs (**a**) and QALYs (**b**). Sensitivity analyses to determine the effect on downstream costs (**a**) and QALYs (**b**) when model inputs were varied across plausible ranges (Table 1). The model inputs represented on the y-axis are probabilities. Wider bars indicate greater variability in estimated downstream costs or QALYs when the input was varied across its plausible range (i.e., the model was most sensitive to uncertainty around these inputs). **a** incorporates one threshold analysis in which we asked what price per student would result in ParentCorps no longer being cost saving

In one-way and multi-way sensitivity analyses, results regarding costs and benefits were highly stable, with ParentCorps remaining cost saving and improving health, even when varying nearly every model assumption across its plausible range. The diagrams depicted in Fig. 2 report the change in the difference in cost (Fig. 2a) or QALYs (Fig. 2b) between ParentCorps enhanced pre-k programming versus standard pre-k programming when each of the model inputs was varied across its plausible range.

#### Sensitivity analyses effect on cost (Fig. 2a)

ParentCorps was cost saving under all circumstances, except under the scenario in which there were fewer than 10 pre-k students per school. The magnitude in cost difference between ParentCorps enhanced pre-k versus standard pre-k ranged from $-10,297 (ParentCorps is cost saving) to $ + 53,062 (ParentCorps is cost generating) when the number of pre-k students per school was varied from 100 to 1 student, respectively. Threshold sensitivity analysis found that ParentCorps would no longer be cost saving if the cost per student exceeded $6400 (>7 times the current cost estimate). ParentCorps still saved money if implemented in schools where only 10% of pre-k students had low levels of self-regulation (more typical of low poverty schools), when the pre-k program served at least 20 students.

When we varied the cost of ParentCorps, the prevalence of low self-regulation in pre-k and the number of students enrolled in the pre-k program, we found that even in the unlikely scenario in which the capacity building investment would last only 1 year instead of 5 years, intervention would continue to be cost saving in schools with more than 40 pre-k students, assuming that 35% of the students had low self-regulation; or in schools with 76 or more students, assuming that more than 15% had low self-regulation.

#### Sensitivity analyses effect on QALYs (Fig. 2b)

When all data inputs were varied across their plausible ranges, ParentCorps consistently increased QALYs, with magnitude ranging from 0.12 to 0.37 QALYs. The benefit originated principally from decreased behavior problems, obesity, diabetes, and drug abuse along with their health sequelae. In threshold analyses, there was no circumstance under which ParentCorps did not improve health and QALYs.

## Discussion

ParentCorps delivered as an enhancement to pre-k programs in high-poverty, urban schools yields meaningful and sustained benefits across academic, behavior, and health domains through age 8. A mathematical model estimates that ParentCorps would save $4387 per person over the life course. This estimate can be considered relative to cost savings figures generated by the Washington State Institute for Public Policy for two public health and preventive family-centered, early childhood interventions with the greatest benefits (i.e., Nurse Family Partnership = $8988; Parents as Teachers = $6638) as well as four parenting interventions for childhood behavior problems with the greatest cost savings (i.e., Triple P Positive Parenting Program = $2201; Parent-child Interaction Therapy = $1704; Parent Management Training-Oregon Model = $1234; and Incredible Years Parent Program = $1039) [47]. The projected cost savings and increased quality-adjusted life expectancy are primarily attributable to ParentCorps’ benefits for children at highest risk for problems based on entering pre-k with low levels of self-regulation. Benefits for this subgroup include impact on childhood obesity and predicted subsequent development of diabetes, and impact on childhood behavior problems and predicted subsequent interaction with the judiciary system, drug abuse, and unemployment. Notably, results underestimate the potential cost savings for the population of children enrolled in pre-k in high-poverty schools, and especially for this subgroup of children with low levels of self-regulation behavior because we did not model the cost savings in childhood and adolescence of decreased need for academic remediation, special education services or mental health services.

The long-term health benefit of ParentCorps delivered as an enhancement to pre-k programming in high-poverty, urban schools (potentially reaching all children enrolled in pre-k) was estimated to be 0.27 QALYs per person. For comparison, the United States Preventive Services Task Force recommends the universal screening of all newborns for phenylketonuria (PKU) and congenital hypothyroidism; these interventions are associated with adding 0.003 QALYs per person [48].

The most influential factors affecting the improvement in predicted QALYs for children in schools with ParentCorps, as tested in sensitivity analyses (Fig. 2b), were the prevalence of low self-regulation, the likelihood of children with low self-regulation developing obesity, the impact of ParentCorps on preventing obesity, and the likelihoods of health sequelae of diabetes and obesity in adulthood. Based on the assumptions in our model, early childhood family-centered interventions embedded in high-poverty schools, such as ParentCorps, that are successful in reaching, engaging and effectively supporting all families, especially the substantial subgroup of families of children with low self-regulation in pre-k, are likely to result in long-term population-level health benefits and cost savings.

Based on the potential for impacting three critical domains of child development (learning, behavior and health), and 8 years of implementation experiences outside of the RCTs, ParentCorps is currently being scaled in New York City (NYC) in partnership with the local Department of Education and the state Office of Mental Health. Although the intervention costs considered in the current study are based on experiences in schools within the RCTs as well as more recent implementation experiences outside of the trials (since 2009), capacity building and recurring implementation costs may increase or decrease as part of implementation in the context of a larger city-wide effort to provide high-quality pre-k programming at the population-level. As part of the implementation process, and in collaboration with city and state partners, capacity building and implementation costs will be calculated when implemented at scale. The impacts on childhood behavior, obesity and academic achievement considered in the current study simulation are based on outcomes from the two RCTs. As part of the ParentCorps strategic growth plan, two hybrid effectiveness/implementation randomized controlled trials (with more than 100 pre-k programs) are underway that will lead to a wealth of information on implementation quality and impact on children and families. At the completion of these studies, we plan to carry out a second cost-effectiveness study based on newly calculated implementation costs and outcomes when delivered at scale in schools serving diverse student populations. In NYC, there are more than 1850 pre-k programs serving nearly 70,000 4-year-olds annually. Approximately half of these programs can be considered high-poverty. As one strategy to reach these pre-k programs, Brotman and colleagues have created a series of professional learnings for principals and teachers to support adoption of ParentCorps evidence-based strategies [49]. These professional learnings are also being studied in the context of randomized controlled trials that will consider costs and outcomes from ParentCorps professional learning relative to ParentCorps programming (as implemented in the original trials) and relative to other professional learning provided by the school district.

Our study has several limitations. An important limitation is that our findings represent the results of a simulation model which is constrained by the limitations of all such models. Most notably, the results depend on the data inputs which are derived from the mean values and plausible ranges from the best available evidence identified by the authors at the time of the study. For example, the variance of the impact of ParentCorps on preventing behavior problems and obesity may have been underestimated because estimates were derived from studies in which a small number of schools were randomized to each intervention condition. However, sensitivity analyses explored considerably smaller impacts and still found the model to be robust. In addition, all base-case childhood inputs were based on data representing the demographics of urban areas in the US, and therefore may not be generalizable to other populations. Although most assumptions were conservative, biased toward finding that ParentCorps was expensive and/or ineffective, the assumption that the likelihood of developing cardiovascular disease was the same in adults with either obesity or diabetes possibly overestimates the likelihood of developing cardiovascular disease in adults with obesity who do not have diabetes. A further limitation is that there are no longitudinal follow-up studies documenting the lifelong effect of early childhood interventions for obesity. Rather, we needed to make assumptions based on the cascading effect of shorter-term benefits of early childhood interventions persisting over time. Finally, to more fully capture the potential cost savings and impact of early intervention, future studies should consider more than one Markov state for childhood to account for new influences as children transition through adolescence, estimate cost savings related to educational and mental health services in childhood and adolescence, and consider the effect of graduation from high school on the likelihood of interacting with the judiciary system, employment and poor health.

## Conclusion

When delivered as an enhancement to pre-k programs in high-poverty, urban schools, ParentCorps results in robust and sustained benefits in learning, behavior and health, especially among children with low levels of self-regulation early in life. Based on mathematical modeling, ParentCorps was estimated to save $4387 per individual and increase each individual’s quality-adjusted life expectancy by 0.27 QALYs. A systematic series of studies is currently underway to consider benefits and costs when ParentCorps is implemented at scale in high-poverty schools serving even more diverse student populations. ParentCorps has the potential to be both cost saving and health generating under nearly all assumptions, and holds promise as a population health approach with substantial return on investment.

## Appendix

**Table 2:** ParentCorps Costs [2005-2014]

	Year 1	Year 2	Year 3+
Training	15 trainees	3 new trainees	2 new trainees
ParentCorps FUNdamentals (4-day) + Program Training (1 day for Friends School/3 days for Parenting Program) (56 h training +16 h prep)	$12,000	$4000	$3000
Manuals & handouts	$2000	$975	$975
School Staff Stipends	$5760	$1080	$1080
Total - Training	19,760	$6055	$5055
Implementation Materials	2 group series	3 group series	3 group series
Brochures, Guides	$1500	$1500	$1500
ParentCorps Program Materials	$3600	$3850	$3850
Equipment	$2000	$0	$0
Healthy Meals	$5830	$6850	$6850
School Staff Time (14 weeks x 3 h/week at per session rates)	$11,000	$11,000	$11,000
Total Implementation Materials	$23,930	$23,200	$23,200
Coaching	2 group series	3 group series	3 group series
Coaching for High-Quality Program Implementation	$60,500	$60,500	$11,500
Grand Total	$104,190	$89,755	$39,755

NOTE: Costs are calculated including data from 2005 to 2014 for capacity and implementation of ParentCorps in a large school (72 students per year; 4 classrooms). The costs below include capacity building and implementation. Capacity building costs includes ParentCorps FUNdamentals, and Training and Coaching for high-quality ParentCorps program implementation. Implementation assumes 2 series of the Parenting Programs in the first year (1 program delivered during the school day and 1 program during after-school hours with a parallel program for pre-k students) and 3 series in the second year and beyond (2 programs during the school day and 1 program during after-school hours). Each program serves 15 to 20 families. Implementation costs include materials, meals and school staff time for after-school programming and coaching. Implementation costs in year three and beyond are consistent with year two with the exception of reduced coaching time and costs

## Abbreviations

pre-k: Pre-kindergarten
QALYs: Quality adjusted life years
RCTs: Randomized controlled trials
